# Restoration of soil quality of degraded grassland can be accelerated by reseeding in an arid area of Northwest China

**DOI:** 10.3389/fpls.2023.1101295

**Published:** 2023-06-13

**Authors:** Qi Lu, Hongbin Ma, Yao Zhou, Jindi Liu, Yan Shen

**Affiliations:** ^1^ Key Laboratory for Model Innovation in Forage Production Efficiency, Ministry of Agriculture and Rural Affairs, Ningxia University, Ningxia, China; ^2^ Ningxia Grassland and Animal Husbandry Engineering Technology Research Center, Ningxia University, Ningxia, China; ^3^ College of Forestry and Prataculture, Ningxia University, Ningxia, China; ^4^ Key Laboratory for Restoration and Reconstruction of Degraded Ecosystems in Northwestern China of Ministry of Education, Ningxia, China

**Keywords:** ecological restoration, restoration rate, soil quality, native species reseeded, desert steppe

## Abstract

Grassland restoration measures control soil degradation and improve soil quality (SQ) worldwide, but there is little knowledge about the effectiveness of restoration measures affecting SQ in arid areas, and the restoration rate of degraded grasslands to natural restoration grasslands and reseeded grasslands remains unclear. To establish a soil quality index (SQI) to evaluate the effects of different grassland restoration measures on SQ, continuous grazing grassland (CG) (as a reference), grazing exclusion grassland (EX), and reseeding grassland (RS) were selected and sampled in the arid desert steppe. Two soil indicator selection methods were conducted (total data set (TDS) and minimum data set (MDS)), followed by three SQ indices (additive soil quality index (SQI_a_), weighted additive soil quality index (SQI_w_), and Nemoro soil quality index (SQI_n_)). The results indicated that SQ was better assessed using the SQI_w_ (*R* ^2 ^= 0.55) compared to SQI_a_ and SQI_n_ for indication differences among the treatments due to the larger coefficient of variance. The SQI_w_-MDS value in CG grassland was 46% and 68% lower than that of EX grassland and RS grassland, respectively. Our findings provided evidence that restoration practices of grazing exclusion and reseeding can significantly improve the SQ in the arid desert steppe, and native plant reseeded can accelerate soil quality restoration.

## 1 Introduction

The restoration of degraded land is a worldwide concern. Grazing is the main use way of grassland (Mottet et al., 2017). Grazing land covers about a quarter of the global land area, mostly in arid and semi-arid lands (Gan et al., 2012; Mcsherry and Ritchie, 2013). Unfortunately, overgrazing has resulted in natural grassland soil degradation worldwide (Xie et al., 2020). The Northwest China desert steppe located in an arid and rainless zone has experienced severe soil degradation driven primarily by inappropriate grazing (Liu et al., 2014; Yu et al., 2018a). So far, numerous vegetation restoration programs have been implemented worldwide to reduce land degradation. In the context of “sustainable intensification”, to improve soil conditions and restore the ecosystem, China has implemented the Returning Grazing Land to Grassland Project (abbreviated as “Grassland Conservation”) (Liu et al., 2008; Ministry of Forestry, 2014). The main measures for engineering the return of cultivated land to grassland in Northwest China include abandonment of cropland, grazing exclusion, and reseeding with local vegetation species (Shang et al., 2014). Among all the types of vegetation restoration programs studied, degraded grassland management measures are mainly based on natural restoration (e.g., grazing exclusion), but such measures often have long restoration cycles and unstable implementation effects (Zhang et al., 2020). Grazing exclusion occurs through natural succession, while reseeding occurs through the restoration of the target community (Bakker et al., 2007; Li et al., 2009). Reseeding is one of the important measures for the restoration of degraded grasslands (Necpálová et al., 2013). Grassland restoration be accelerated by reseeding native species, and grassland ecosystem functioning and soil quality can be restored (Wang et al., 2023). Therefore, for example, *Agropyron mongolicum* Keng., *Lespedeza potaninii* Vass., and *Astragalus melilotoides* have been used widely in the restoration project of degraded grasslands in arid areas of Northwest China (Xie et al., 2015).

Degraded grassland management practices are a major factor affecting soil quality (SQ) and soil productivity sustainability (Raiesi, 2017). Maintaining good vegetation to maintain or improve water and air quality is the premise of sustainable utilization of soil (Karlen et al., 1997). SQ indicators are strongly related to land-use type and management factors (Liu et al., 2018; Valle and Carrasco, 2018). Many studies focused primarily on the restoration management of individual soil physical properties (Yu et al., 2014) or soil nutrients (Deng et al., 2018). However, the interaction among soil properties and their response to grassland restoration management is complex and responds differently to management practices (Raiesi, 2017). The single evaluation of several different soil properties may complicate the interpretation of the results considerably. Reseeding of legumes increased nitrogen effectiveness and improved grassland biodiversity and plant community biomass when compared to grazing sites (Li et al., 2015). However, other studies have shown that soil disturbance during reseeding may stimulate organic matter decomposition leading to a reduction in soil nutrients (Conant et al., 2007). To fully understand the effects of reseeding on the recovery of degraded grasslands, it is necessary to combine soil properties into an overall single index of perception to assess these relationships, which may make the assessment more meaningful and practical. A reliable and exact SQ assessment is essential to evaluating soil deterioration and restoration potential in the desert steppe (Yu et al., 2018b). However, little attention is currently being paid to the impact of restoration measures (e.g., grazing exclusion and reseeding) on SQ. Therefore, the effect of the conversion from continuous grazing grassland to grazing exclusion and reseeding grassland on SQ has yet to be quantified in arid areas of Northwest China.

SQ must be evaluated by several soil properties due to high variability in soil properties and function (Andrews et al., 2004). For the comprehensive evaluation of SQ, the soil quality index (SQI) has been widely used at various scales and locations because of its flexible quantification and convenience to use (Qi et al., 2009; Bretzel et al., 2016; Yu et al., 2018b). As an effective tool for selecting the most important soil properties and dimension reduction, principal component analysis (PCA) is widely used for defining the minimum data set (MDS) (Andrews et al., 2002a). Generally, the MDS method could select the indicators that best represent SQ and reduce data redundancy and should fully represent the total data set (TDS) (Cheng et al., 2016). Meanwhile, an MDS reduces subjective anthropogenic interference and the time and cost of the SQ evaluation (Rezaei et al., 2006; Zhang et al., 2016). After the soil indicators in the MDS are determined, the data need to be normalized with scoring function (linear and non-linear scoring), and the non-linear scoring method was considered the superior method to scoring SQ indicators to the linear method (Andrews et al., 2002a). The integration of dimensionless indicators (obtained after normalization by the scoring functions) into SQI is possible through many procedures based on the additive soil quality index (SQI_a_), the weighted additive soil quality index (SQI_w_), and Nemoro soil quality index (SQI_n_) approaches (Askari and Holden, 2014; Rahmanipour et al., 2014; Zhang et al., 2016; Nabiollahi et al., 2017; Nabiollahi et al., 2018). The additive soil quality index (SQI_a_), the weighted additive soil quality index (SQI_w_), and the Nemoro soil quality index (SQI_n_) are used to integrate dimensionless indicators into the mass index (Nabiollahi et al., 2018). The SQI_a_ is a summation of the scores of indicators (Doran and Parkin, 1994). SQI_w_ takes into account the importance of each indicator and specifies the weight of each indicator in the score indexing process (Nabiollahi et al., 2017). SQI_n_ emphasizes the influence of soil quality constraints without considering their weights (Qi et al., 2009; Rahmanipour et al., 2014). Although SQIs are effective methods to reflect SQ changes in the conversion of land uses, there is little available information on SQ evaluation along with the conversion from continuous grazing grassland to grazing exclusion and reseeding grassland in arid areas of Northwest China.

The responses of SQ to different grassland restoration managements are of great significance for improving the sustainable development of grassland. Nevertheless, there are few studies on SQ changes during the transformation of degraded grassland (i.e., overgrazing grassland) to grazing exclusion grassland and reseeded grassland in the desert steppe, and the restoration rate of degraded grasslands to natural restoration grasslands and reseeded grasslands remains unclear. The SQ monitoring provides an opportunity to evaluate the effect of grassland restoration management, and it is important to choose appropriate desert steppe restoration management in fragile regions. In this study, we hypothesized that the improvement of SQ would be promoted more quickly by artificial restoration management (e.g., reseeding and reestablishment) compared with grazing exclusion management. Thus, the purposes of this study were as follows: 1) to investigate the effects of grazing exclusion and reseeding on degraded desert steppe soil physical and chemical properties, 2) to identify the most appropriate integration procedure (SQI_a_, SQI_w_, and SQI_n_) for different restoration managements in the desert steppe, and 3) to quantify the SQ under the natural and artificial restoration managements of the degraded desert steppe and provide pertinence and theoretical basis for the rational management and soil restoration.

## 2 Materials and methods

### 2.1 Study area

**Figure 1:**
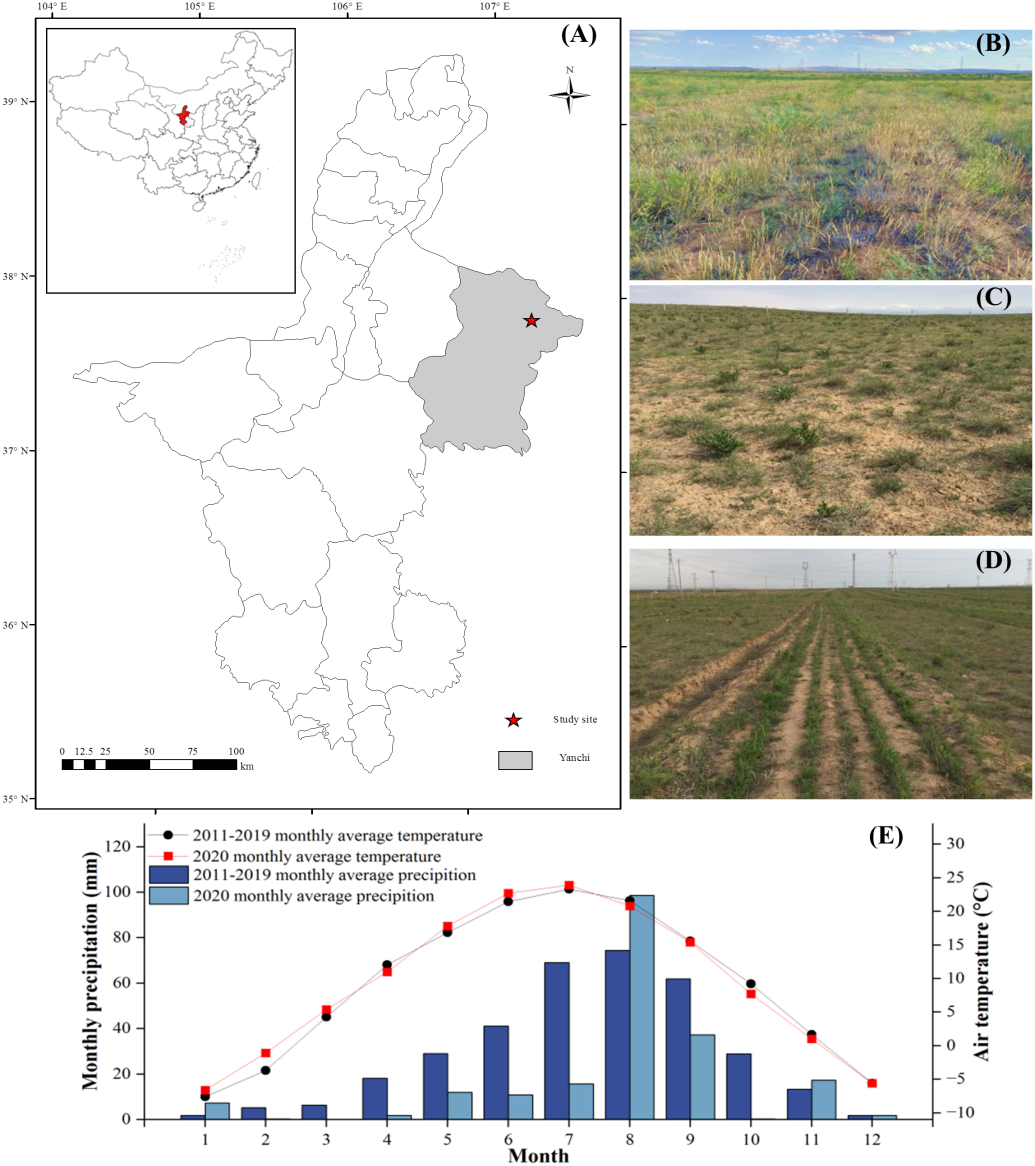
**(A)** Location of the study area in desert steppe. **(B)** Grazing exclusion grassland. **(C)** Continuous grazing grassland. **(D)** Reseeded grassland. **(E)** Air temperature (filled lines) and precipitation (bars) recorded in 2011-2020 at the study site.

The study was conducted in Yanchi County (37°28′–37°29′N; 106°56′–106°57′E), Dashuikeng Town, located in the Mu Us Desert in Northwest China (**Figure 1**). This area has a temperate continental monsoon arid climate, with an average annual temperature of 7.6°C and mean annual precipitation of 290 mm. The potential evaporation is approximately 2,132.0 mm. The frost-free period is approximately 162 days. The soil type in this region is mainly dominantly desertification sierozem, slightly alkaline, classified based on USDA Soil Taxonomy (Soil Survey Staff, 2014). The zonal vegetation is the desert steppe. The most common natural grassland-dominated species include *A. mongolicum* Keng., *Leymus secalinus* (Georgi) Tzvel., *Aster altaicus* Willd., *L. potaninii* Vass., *Stipa bungeana* Trin., *Pennisetum flaccidum* Grisebach., and *Artemisia scoparia* Waldst. et Kit.

The study site is 200 ha of open and flat natural grassland. In the early 1990s, it was grazed continuously by Tan sheep, but the characteristics of soil and vegetation cover were relatively uniform. The restoration project was started in 2002, and the exclusion project was established in 2002. In the initial livestock removal process, the dominant plant species in the whole area were grasses and legumes.

### 2.2 Experimental design and soil sampling

Three land-use types were established in this study, including continuous grazing grassland (CG) (100 ha), grazing exclusion grassland (EX) (20 ha), and reseeded grassland (RS) (55 ha) (**Figure 1**). The land uses in the study area have similar slope aspects and parent materials. Before 2002, the permanent grassland was mainly used as grazing grassland, and overgrazing led to grassland degradation. To protect the natural grassland and restore the degraded overgrazing grassland, grassland grazing is excluded all year round, but some continue grazing. The detailed information about these grassland-use treatments is as follows: 1) CG is a grazing regime of continuous grazing throughout the year, and the annual stocking rate has been 3–3.5 sheep ha^−1^ since 1982. 2) EX was started in the year 2002. Before grazing exclusion, the site was used as grazing land as that of the CG grassland. 3) RS reseeded native perennial grass species based on grazing exclusion in May 2017. According to the previous survey of the nearby non-degraded grassland, the reseeding amount was determined according to the dominant plant species density in the non-degraded grassland. Seeds of local ecotypes were produced from the arid areas of Northwest China. According to the vegetation survey data of nearby non-degraded grasslands, the ratio of Gramineae to legumes is 6:4. The RS grassland was seeded with 2.5 kg ha^−1^ of three native plant seed mixtures. The seed mixtures contained, by weight, 60% K. *A. mongolicum*, 20% V. *L. potaninii*, and 20% P. *A. melilotoides*. The reseeded holes were 3 cm deep and spaced 20 cm apart. The vegetation coverage measures 33% before the reseeded treatment.

In early August 2020, within each grassland-use treatment site, three distinct replicate plots (20 × 20 m) were established for sampling. The distance between any two plots was >2 km to ensure that the repetitions were representative. All plots were surrounded by a buffer zone (6–8 m) to avoid the influence of the edge on adjacent plots. Within each plot, five subplots (1 m × 1 m) were randomly established, at the four corners and the center, investigating the dominant grass species, height, above-ground biomass, density, geographical location, and altitude (**Table 1**). After the above-ground biomass and litter were removed, five soil samples from subplots were randomly collected with a soil core sampler (4.0-cm diameter) from each 0–20-cm layer in each plot and mixed thoroughly to produce a composite sample. The soil samples were air-dried at room temperature, and the root materials and other visible debris were removed and passed through a 2-mm sieve before the following chemical and physical analyses. Five undisturbed soil samples were collected at a depth of 0–20 cm with 100-cm^3^ corers, which were used to determine soil bulk density, porosity, and soil holding capacity. At the same sampling points, the same number of undisturbed soil samples were obtained at depths of 0–20 cm and then sealed in a plastic box to avoid being squeezed and impacted during transportation back to the laboratory for the determination of soil aggregate indicators.

**Table 1:** Basic information of experimental plots.

Grassland uses	CG	EX	RS
Position coordinates	N37°28′58″	N37°29′37″	N37°29′15″
	E106°57′37″	E106°56′35″	E106°56′05″
Vegetation coverage (%)	44.40 ± 2.96a	49.00 ± 2.72a	47.00 ± 1.35a
Above-ground biomass (g·m^−2^)	35.51 ± 1.16b	37.62 ± 4.46b	58.61 ± 1.69a
Mean vegetation height (cm)	5.77 ± 0.12c	8.67 ± 0.19b	10.47 ± 0.67a
Vegetation density (plant·m^−2^)	73.20 ± 3.85c	132.33 ± 3.38b	149.00 ± 5.69a
Restored years	0 (not restored)	18	3

Results are shown as the mean ( ± SD).

CG, continuous grazing grassland; EX, grazing exclusion grassland; RS, reseeded grassland.

### 2.3 Soil analyses

To characterize the study area as a whole, 120 samples were collected from the soil layer (0–20 cm). Twenty soil properties were measured for each sample (**Table 2**): porosity, water holding capacity (WHC), granulometric analysis (sand, silt, and clay percentage), soil bulk density (BD), pH, electrical conductivity (EC), cation exchange capacity (CEC), geometric mean diameter (GMD), mean weight diameter (MWD), soil organic carbon (SOC), total nitrogen (TN), total phosphorus (TP), available nitrogen (AN), available phosphorus (AP), and available potassium (AK).

**Table 2:** The methods and references for laboratory analysis of SQI used in the study.

Soil properties	Analytical methods	Reference
Soil bulk density (BD)	Cutting ring water immersion method	Zhang et al., 2021
Soil porosity, capillary porosity, and non-capillary porosity		
Water holding capacity (WHC)	Gravimetric with oven drying method	Gong et al., 2015
Granulometric analysis (sand, silt, and clay percentage)	MasterSizer 2000 method	Deng et al., 2016
pH	Soil/water solution of 1:5	Yu et al., 2014
Electrical conductivity (EC)	Soil/water solution of 1:5	Yu et al., 2014
Cation exchange capacity (CEC)	Ammonium acetate at pH 7	USDA, 1996
Geometric mean diameter (GMD)	Wet sieving method	Kemper and Rosenau, 1986
Mean weight diameter (MWD)		
Soil organic carbon (SOC)	Potassium dichromate oxidation	Zhu et al., 2014
Total nitrogen (TN)	Automatic Kjeldahl method	Liu and Jiang, 1996
Total phosphorus (TP)	Wet digestion with sodium hydroxide	Smith and Bain, 1982
Available nitrogen (AN)	NaOH hydrolysis	Stanford, 1982
Available phosphorus (AP)	Sodium bicarbonate	Olsen, 1954
Available potassium (AK)	Ammonium acetate	Thomas, 1982

SQI, soil quality index.

The dry-sieving macroaggregate (DSMA) was calculated using the following equation:


$$\text{DSMA}\;\left(\%\right)=\frac{M_{r}}{M_{t}}\times 100,$$

where *M_r_* is the mass of dry-sieving aggregates >0.25 mm (g) and *M_t_* is the total mass of the dry-sieving soil (g).

The MWD and GMD of soil aggregates were calculated by the following equation (Kemper and Rosenau, 1986):


$$\text{MWD}=\sum_{i=1}^{n}X_{i}W_{i},$$

$$\text{GMD}=exp\left(\frac{\sum_{i=1}^{n}\left(lnX_{i}\right)W_{i}}{\sum_{i=1}^{n}W_{i}}\right),$$

where *X_i_* is the mean diameter of each size fraction (mm) and *W_i_* is the proportion of the total sample mass in the corresponding size fraction after deducing the stone mass as indicated above.

The soil erodibility (K value) was calculated by the following equation (Dou et al., 2020):


$$\text{K}=7.954\times \left\{0.0017+0.0494\times \text{exp}\left[-0.5\times {\left(\frac{\text{logGMD}+1.675}{0.6989}\right)}^{2}\right]\right\},$$

where *GMD* is the geometric mean diameter, and 7.954, 0.0017, 0.0494, 1.675, and 0.6989 are the constant terms.

### 2.4 Soil quality index assessment

#### 2.4.1 TDS and MDS

TDS and MDS were used to determine the appropriate indicators. First, 19 properties were analyzed by one-way analysis of variance (ANOVA) to evaluate the effects of different grassland use on soil properties. Second, the properties with a significant difference (*p*< 0.05) between the three land uses were selected as the TDS indicators for formulating the SQ indices. Third, PCA was applied to the standardized data matrix of the TDS to reduce data redundancy and determine the most important indicators of the MDS (Raiesi, 2017). Only the PCs with eigenvalues ≥ 1 and those that explained at least 5% of the variation in the data were deployed to identify the MDS (Sharma et al., 2005). However, if all the properties with weighted absolute values within 10% of the highest indicator value for each PC were selected, then the MDS will lead to data verbosity. According to Andrews and Carroll (2001), the use of PCA to calculate the factor load usually only considers the load of a certain indicator on one PC, and there will be lost information on the indicator on other PCs with eigenvalues ≥ 1 (Yemefack et al., 2006). To avoid lengthy data and loss of important information, this defect can be overcome by calculating the norm value (vector norm) of the variable. The geometric meaning of the norm value is the magnitude (length) of the vector norm of the variable in the multi-dimensional space composed of PCs. The higher the norm value, the greater the comprehensive load of the variable on all PCs, and the stronger the explanatory power of variables to the overall SQ information. Norm value was calculated using the following equation (Chen et al., 2013):


$$N_{ik}=\sqrt{\sum_{i}^{k}(U_{i}^{k2}\lambda_{k})},$$

where *λ_k_* is the eigenvalue of the PC and *U_ik_* is the loading of soil variable *i* on the PC*_k_*. Indicators receiving *N_ik_* within 10% of the highest norm values were selected for the MDS.

#### 2.4.2 Indicator scoring and weighting the MDS indicators

After the indicators of TDS and MDS were determined, the non-linear scoring function transforms the soil indicators into unit-less scores within 0–1. The sigmoidal function was applied (Andrews et al., 2002b) as follows:


$$\text{SNL}=\frac{a}{1+{\left(x/x_{0}\right)}^{b}},$$

where *a* is a maximum score equal to 1 in this study, *x* is the soil variable value, *x*_0_ is the mean value of the variable, and *b* is the slope assumed to be −2.5 for “more is better” functions and +2.5 for “low is better” ones (Yu et al., 2018b).

#### 2.4.3 Developing and validating soil quality indices

For TDS and MDS, the transformed indicator scores were integrated into three SQIs, including the SQI_a_ (Andrews et al., 2002b), SQI_w_ (Askari and Holden, 2014), and SQI_n_ (Qi et al., 2009), as follows:


$$SQI_{a}=\frac{\sum_{i}^{n}N_{i}}{n},$$

$$SQI_{w}=\sum_{i=1}^{n}W_{i}N_{i},$$

$$SQI_{n}=\sqrt{\frac{Pave^{2}+Pmin^{2}}{2}}\times \frac{n-1}{n},$$

where *N_i_* is the non-linear indicator score, *n* is the number of indicators, *W_i_* is the weighting factor for the soil indicator derived from the factor analysis, *Pave* is the average, and *Pmin* is the minimum of the scores of the selected indicators at each sampling point. Higher SQI values mean better soil function and soil process and reflect the positive effects of grassland restoration measures.

#### 2.4.4 Stage increase rate of SQI

The restoration rate of the SQI was computed as follows (Zhang et al., 2012; Guo et al., 2018):


$$R_{SQI}=\text{ΔSQI}/\left(\text{Δt}\times SQI_{ref}\right)\times 100,$$

where *ΔSQI* refers to SQI at the start and end of a recovery stage and *Δt* represents the restoration time for each restoration type. *SQI_ref_* refers to SQI for continuous grazing grassland.

### 2.5 Statistical analysis

ANOVA and least significant difference (LSD) were used to assess statistically significant differences (*p*< 0.05) under different grassland uses. The correlation between soil indicators and SQIs was analyzed by using Pearson’s correlation. The PCA method was used to select the most suitable soil indicators for evaluating SQ. Before statistical analysis, the normality and equal variance of all data sets were tested to meet the assumptions of statistical analysis. All statistical analyses were conducted using Microsoft Excel 2010 and SPSS 20.0 (IBM, USA) software.

## 3 Results

### 3.1 Changes in measured soil physical and chemical properties

**Figure 2:**
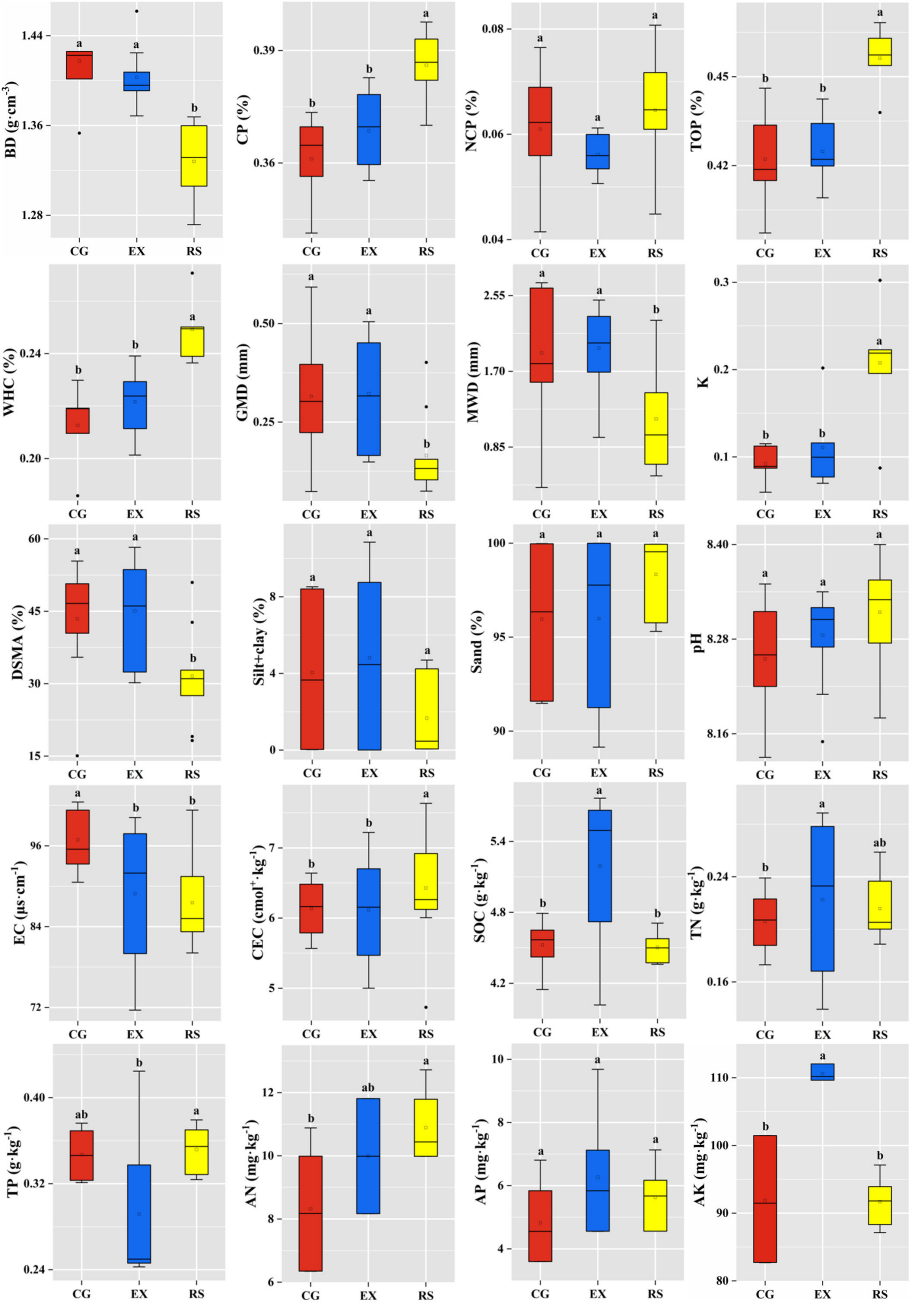
Soil indicators measured as potential soil quality indicators of desert steppe under different land-use types. CG, continuous grazing grassland; EX, grazing exclusion grassland; RS, reseeded grassland; BD, soil bulk density; CP, capillary porosity; NCP, non-capillary porosity; TOP, total porosity; WHC, water holding capacity; GMD, geometric mean diameter; MWD, mean weight diameter; K, the soil erodibility; DSMA, dry-sieving macroaggregate (>0.25 mm); pH, soil reaction; EC, electrical conductivity; CEC, cation exchange capacity; SOC, soil organic carbon; TN, total nitrogen; TP, total phosphorus; AN, available nitrogen; AP, available phosphorus; AK, available potassium. The same lowercase letters within the grassland uses indicate not significantly different (*p*< 0.05).

No remarkable difference was found for NCP, silt+clay, sand, and pH among the three grassland-use types (**Figure 2**). Furthermore, 16 soil indicators (namely, BD, CP, TOP, WHC, GMD, MWD, K, DSMA, EC, CEC, SOC, TN, TP, AN, AP, and AK) differed significantly (*p*< 0.05); thus, they were selected as values of the TDS for PC among the three grassland uses.

Significantly lower BD, GMD, MWD, and DSMA content were observed in RS compared with the EX and CG (*p*< 0.05). However, a similar trend was observed for the GMD, MWD, and DSMA content, which ranked as EX > CG > RS, and EX and CG were not significantly different (*p* > 0.05) (**Figure 2**). RS treatment had significantly higher CP, TOP, WHC, K, and CEC values than EX and CG (*p*< 0.05), yet there were no significant differences in CP, TOP, WHC, K, and CEC values between EX and CG (*p* > 0.05). The highest SOC, AP, and AK values were observed in EX soils (*p*< 0.05). The highest and lowest TN content were observed in EX and CG. The positive effects of RS on TP and AN were significantly stronger than those on EX (*p*< 0.05).

### 3.2 Indicator selection for MDS

The 15 soil quality properties considered in the PCA were grouped into components. According to the results of this study, the first four PCs had eigenvalues > 1, each explaining at least 5% of the data variation and accounting for 94.1% of the data of total variance (**Table 3**). Soil property communalities indicated that the MDS can replace the full data set for the soil quality evaluation of different grassland uses.

**Table 3:** Results of principal component analysis (PCA) of the total data set and the three soil processes.

Principal component	PC1	PC2	PC3	PC4	Norm value	Group
BD (g·cm^−3^)	**−0.819**	0.044	−0.342	0.376	4.629	1
CP (%)	0.565	**−0.590**	0.431	0.119	4.001	2
TOP (%)	**0.797**	−0.272	0.392	−0.282	4.727	1
WHC (%)	0.749	−0.374	**0.507**	−0.104	4.661	3
GMD (mm)	−0.732	0.354	**0.533**	−0.128	4.488	3
MWD (mm)	**−0.847**	0.136	0.417	0.218	4.975	1
K	**0.813**	−0.353	−0.442	0.031	5.097	1
DSMA (%)	**−0.800**	0.322	0.460	−0.174	4.933	1
EC (μs·cm^−1^)	0.000	**0.890**	−0.198	−0.003	3.812	2
CEC (cmol^+^·kg^−1^)	**0.581**	0.741	−0.008	−0.160	4.755	1
SOC (g·kg^−1^)	0.397	**0.744**	0.287	0.388	3.955	2
TN (g·kg^−1^)	0.485	**0.746**	0.056	0.423	4.357	2
TP (g·kg^−1^)	0.442	**0.875**	−0.002	−0.154	4.880	2
AN (mg·kg^−1^)	0.730	0.097	0.207	**0.597**	3.955	4
AK (mg·kg^−1^)	−0.320	−0.749	0.100	**0.515**	3.669	4
Eigenvalue	6.299	4.725	1.756	1.342		
Percent	41.997	31.497	11.708	8.944		
Cumulative percent	41.997	73.494	85.202	94.146		

The data in bold indicate highly weighted variables.

BD, soil bulk density; CP, capillary porosity; TOP, total porosity; WHC, water holding capacity; GMD, geometric mean diameter; MWD, mean weight diameter; K, the soil erodibility; DSMA, dry-sieving macroaggregate (>0.25 mm); EC, electrical conductivity; CEC, cation exchange capacity; SOC, soil organic carbon; TN, total nitrogen; TP, total phosphorus; AN, available nitrogen; AK, available potassium.

**Figure 3:**
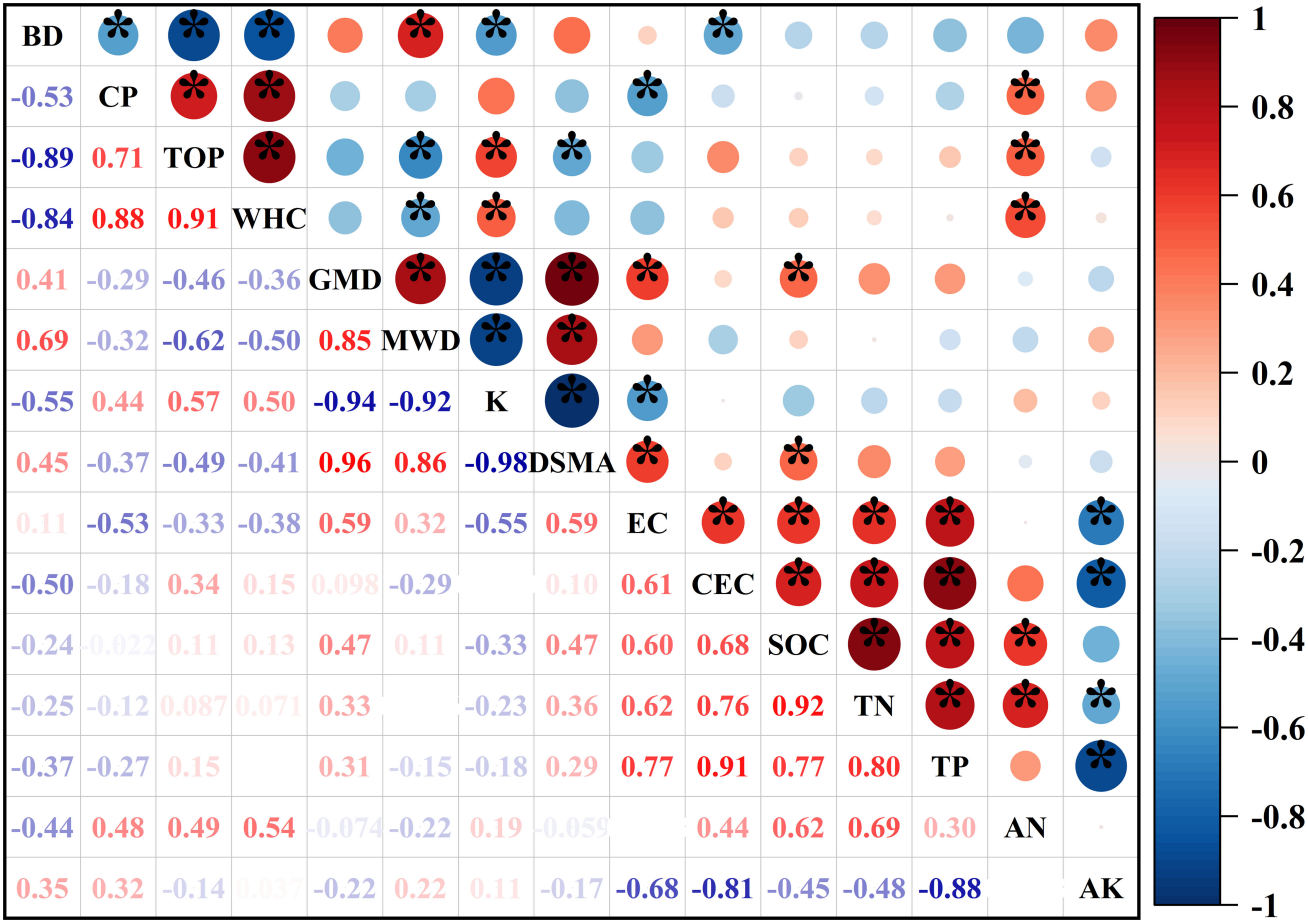
Pearson’s correlation coefficients between soil indicators. Indicated values represent the correlation coefficient. The red color indicates a positive correlation, and the blue color indicates a negative correlation. See **Table 3** for abbreviations. * significant at the 0.05 probability levels.

According to the factor loading (≥0.50), these soil properties were divided into four PCs. If the load of an index in different PCs is >0.5, then the index will be incorporated into a group with a low correlation with other indices. Group 1 included BD, TOP, MWD, K, DSMA, and CEC. The K had the highest norm value (5.10), and the norm values of BD, TOP, MWD, DSMA, and CEC were all within the 10% fluctuation range of the K norm value. A significant positive correlation was found between BD, TOP, MWD, DSMA, and K. No significant correlation exists between K and CEC (**Figure 3**); thus, K and CEC were included in the MDS. Group 2 contained CP, EC, SOC, TN, and TP. The norm value of TP (4.88) is the highest, and no other indicators were within 10% of this value. Therefore, TP, which has the highest norm value, was selected for inclusion in the MDS. Group 3 included WHC and GMD. No significant correlation exists between the two indices, and the norm value of GMD was within the maximum 10% of the norm value of WHC (4.66). WHC and GMD were included in the MDS. Group 4 included two indicators: AN and AK. No significant correlation exists between the two indicators, and the norm value of AK was within 10% of the norm value of the AN (3.96). AN and AK were included in the MDS. Therefore, when combining the PCA and Pearson’s correlation analysis, the refined MDS included the following indicators: K, CEC, TP, GMD, WHC, AK, and AN (**Table 3**; **Figure 3**).

### 3.3 Evaluation of SQIs with TDS and MDS methods

**Figure 4:**
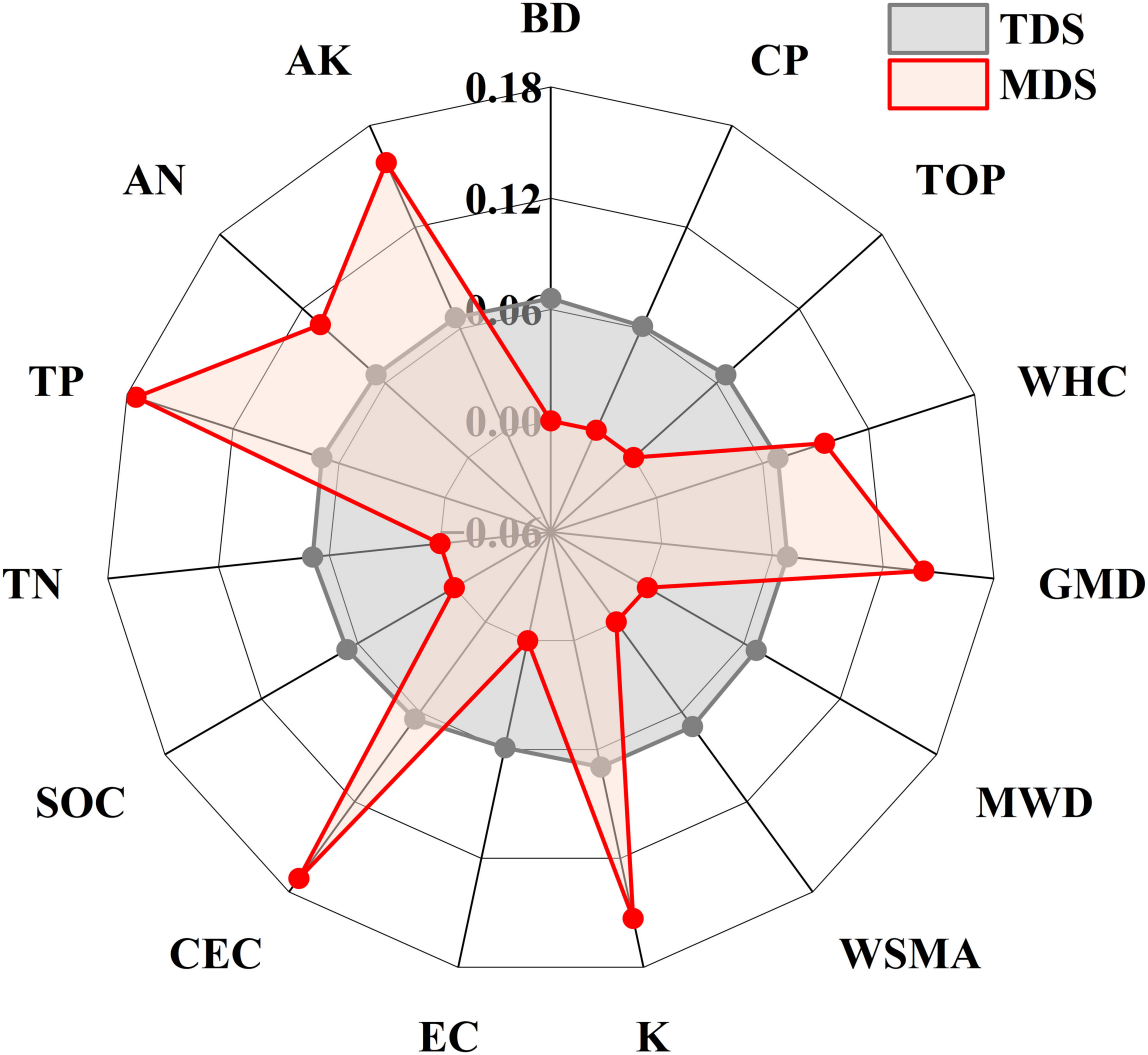
Weight assignment of indicators in TDS and MDS approaches. TDS, total data set; MDS, minimum data set.

In **Figure 4**, PCA was performed for each index of the MDS. K, CEC, TP, GMD, WHC, AK, and AN have a common factor variance and weight. The 15 soil indicators were selected through the PCA for their commonalities and weights in the TDS method. Through PCA for the TDS method, the weights of the different indicators varied lower, with the highest weight for the dry-sieving macoaggregate (DSMA) (0.070) and the lowest weight for EC (0.059). On the contrary, the MDS method was higher. Overall, TP was assigned the highest weight (0.175), and the WHC was assigned the lowest weight (0.095).

**Figure 5:**
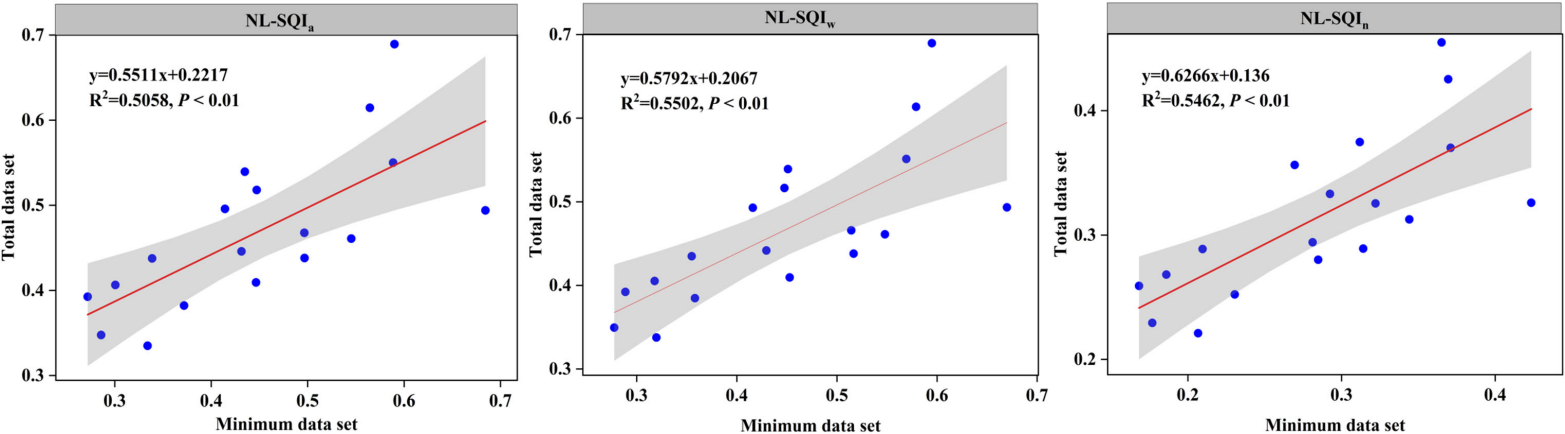
Linear relationship between SQIs (SQI_a_, SQI_w_, and SQI_n_) calculated using the TDS and MDS methods, and non-linear scoring methods.

As a whole, the results of the regression analysis showed that there was a significant correlation between the SQIs (SQI_a_, SQI_w_, and SQI_n_), and the *R* ^2^ value of the SQI_w_ was greater than that of the SQI_a_ and SQI_n_, indicating greater sensitivity (*p*< 0.01) (**Figure 5**). The MDS selected by PCA can quantitatively calculate the changes in SQ when using non-linear scoring. Further, the correlations between SQIs-TDS and SQIs-MDS values were significant, suggesting that the MDS method can better represent the TDS method.

### 3.4 Changes in soil quality under different land uses

**Figure 6:**
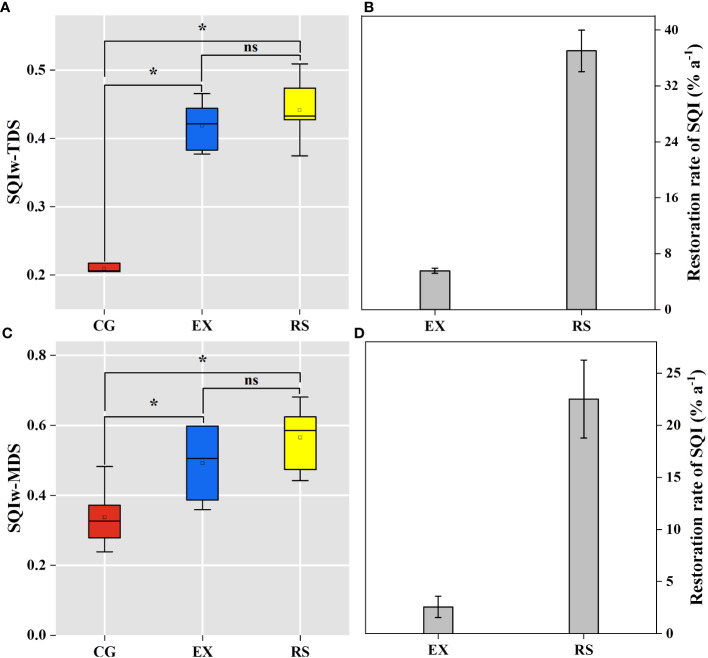
Comparison of the weighted additive soil quality index (SQI_w_) development in this study among different land uses **(A, C)**. Stage recovery rates of the weighted additive soil quality index (SQI_w_) **(B, D)**. CG, continuous grazing grassland; EX, grazing exclusion grassland; RS, reseeded grassland. Error bars correspond to standard deviation. * significant at the 0.05 probability levels (one-way ANOVA) (n = 3). ns, no significant.

In general, The SQI_w_-TDS values (0.42 for EX, 0.34 for CG, and 0.57 for RS) were always greater than SQI_w_-MDS values (0.40 for EX, 0.21 for CG, and 0.44 for RS) in the three different grassland-use types (**Figures 6A, C**). SQ in the study area increased among grassland restoration methods from CG grassland to EX and RS grassland. The results of the two SQIs all showed that SQ under CG treatment was significantly lower than that under the EX and RS treatments (*p*< 0.05), and no significant differences (*p* > 0.05) were found among EX and RS. The RS attained the fastest restoration rate of 3.75% a^−1^, while the EX realized a restoration rate of 1.02% a^−1^ (**Figures 6B, D**).

## 4 Discussion

### 4.1 Soil properties under different grassland uses

Changes in the soil physiochemical properties following grassland-use changes play critical roles in evaluating land recovery in degraded grasslands (Wang et al., 2021). Therefore, our SQI assessment was focused on soil structure and fertility. In relation to soil physical functions, in our study, there are higher BD values in continuous grazing and grazing exclusion sites, compared with those in reseeded sites (**Figure 2**). The enhanced BD in continuous grazing grassland could be explained by the increased soil compaction due to foot trampling by livestock (Byrnes et al., 2018; Benevenute et al., 2020; Botta et al., 2020). Notably, the high BD values in the grazing exclusion grasslands, which could be associated with soil compaction from animal trampling, are not rapidly relieved by natural processes. The different responses could be attributed to the short grazing exclusion duration time (18 years). Previous studies have shown that degradation of soil properties caused by compaction from animal trampling may persist for more than 100 years (Sharratt et al., 1998). This study showed that BD from reseeded grassland was lower than that from the continuous grazing and grazing exclusion grassland uses, while soil CP, TOP, and WHC showed a reverse trend. On the one hand, it was mainly due to the cultivation before the reseeding of the grassland, which could destroy soil structure (Hebb et al., 2017). On the other hand, plant roots change the soil structure, which leads to an increase in soil porosity (Shao et al., 2020). The high WHC content in reseeded sites could be ascribed to the high vegetation coverage that reduces soil evaporation, which can maintain more soil water (Jia et al., 2019). Over the past few decades, the studied native grasslands have been under overgrazing pressure by sheep, drought stress from low rainfall (approximately 290 mm), and high potential evaporation (approximately 2,132.0 mm), which are important factors contributing to WHC loss in continuous grazing grassland.

Primary production is the source of C input into the soil (Li et al., 2022). In the native steppe, selective ingestion by sheep generally leads to a decrease in plant production and a consequent reduction in carbon input. In general, grazing exclusion increased SOC content because grazing exclusion increases carbon input from the decomposition of litter (Li et al., 2022). Interestingly, our results found that reseeded grasslands did not improve the SOC content compared to those in the continuous grazing grassland, but the above-ground biomass of reseeding grassland was significantly higher than that of grazing exclusion grassland (**Table 1**; **Figure 2**). This finding can be explained because of lower soil organic matter input by the corresponding reseeded plant (Wang et al., 2021). In addition, a possible reason is that seed plants need to absorb and consume large amounts of soil nutrients to grow in a short time after reseeding; however, this inference should be further investigated. In our study, two restoration measures, exclusion grazing and reseeding, significantly increased TN and AN contents (**Figure 2**). Previously, it has been observed that the trampling by grazing livestock causes soil consolidation and reduces soil water content, creating poor ventilation conditions, which in turn promotes denitrification and reduces the TN content (Chai et al., 2019). Furthermore, livestock feeding can increase the compensatory growth of the plant, increasing soil TP and TN consumption (Tang et al., 2016). We found that the AN content was significantly enhanced by reseeded measure compared to the grazing exclusion measure (**Figure 2**). Reseeded legume plants can convert gaseous nitrogen to ammonium nitrogen, thereby increasing the amount of nitrogen in the soil that can be absorbed and used by plants (Wang et al., 2021).

Aggregate stability and soil erodibility factors as important indicators of soil quality can reflect the stability of the soil’s physical structure (Dou et al., 2020). The MWD and GMD of soil aggregates play a vital role in assessing the stability of aggregates. Overall, continuous grazing and grazing exclusion showed significantly higher MWD, GMD, and WSMA values in soil aggregate than in reseeded grassland (**Figure 2**). This finding is consistent with the study performed by Wang et al. (2012), who indicated that natural recovery is an effective measure to improve aggregate stability compared with reseeded recovery measures. Accordingly, reseeding creates the risk of reduced soil erosion resistance of the soil, which reduces the stability of soil aggregates (Dou et al., 2020). The lower soil aggregate stability and higher K value in reseeded grassland could be attributed to the following factors. First, the restoration time of reseeded grassland was short, and the surface soil layer was loose due to the weak root system of the reseeded vegetation. Second, when the WHC was high, water would enter into the soil pore space, making the macroaggregates swell with water absorption and then be squeezed, leading to disintegration (Dou et al., 2020). Third, more human interference damages soil structure, leading to an increase in the possibility and sensitivity of soil erosion (Wang et al., 2021).

### 4.2 Selection of MDS indicators

Most soil physical and chemical properties showed statistical differences among desert steppe uses, which indicated that desert steppe uses have an important influence on soil properties (Deléglise et al., 2011; Jian et al., 2015; Deng et al., 2018). The 15 selected soil indicators in response to different land uses did not have consistent results, which is mainly attributed to different desert steppe use types leading to different soil processes and functions, which had complex effects on soil properties (Kooch et al., 2018). Results showed that grazing exclusion and reseeding restoration management affected most soil properties evaluated in this study, except NCP, silt+clay, sand, pH, and AP (**Figure 2**). Through the MDS selection, K, CEC, TP, GMD, WHC, AN, and AK were retained as the most important soil indicators to assess the effects of land-use treatments on SQ in the current study. The selected SQ indicators of MDS are the easiest to measure and are more familiar to soil laboratory specialists and local land users. Overall, these findings indicate the key roles of soil water holding capacity, erodibility, and aggregate stability in determining the impact of grassland-use changes on soil quality. Biological indicators have attracted more attention because they are more sensitive to environmental variability (Shao et al., 2020). Nevertheless, due to the simple analysis methods and the low measurement costs, several previous studies have selected only soil physicochemical properties as indicators for soil quality assessment (Guo et al., 2017; Nabiollahi et al., 2017; Zhang et al., 2019). Many studies reported correlations between soil biological indicators and physicochemical indicators. For instance, soil microbial and enzyme activities are often closely correlated with variations in chemical indicators (soil nutrient content) (Raiesi and Kabiri, 2016). Soil organic matter and biological properties determine the soil’s physical structure (e.g., erodibility and soil aggregate stability) (Yu et al., 2018a). Consequently, some of the variations in the biological indicators of the soil can be explained by soil physicochemical indicators.

### 4.3 Comparison of soil quality indexing methods

TDS has been widely regarded as a comprehensive SQ assessment method. However, such a method presents a dilemma between comprehensiveness and the cost of laboratory analysis for evaluation results (Raiesi, 2017). In contrast, MDS is an effective method to assess SQ because there are fewer data reduplication and great accuracy to quantify the effects of grassland restoration changes on SQ. Consequently, the establishment of an MDS for soil quality evaluation is widely accepted (Lin et al., 2017).

Results indicated that in this study area, the SQI_w_ model was better for SQI computing when compared to the SQI_a_ and SQI_n_ indices. Compared with SQI_n_, SQI_w_ applies weights to key soil indicators, and the scores for each soil indicator were determined independently (Rahmanipour et al., 2014). The major limitation of SQI_n_ can probably be attributed to the fact that the lowest scoring indicator is added to the average scores, thus giving it a higher weighted value (Qi et al., 2009). Qi et al. (2009) focused on agricultural land in China, considering the relationship between SQI_w_ and SQI_n_, indicating that the SQI_w_ (*R* ^2 ^= 0.65) was better than SQI_n_ (*R* ^2 ^= 0.57). Such a result was consistent with that of Rahmanipour et al. (2014) who also assessed that the SQI_w_ (*R* ^2 ^= 0.34) model was better than the SQI_n_ (*R* ^2 ^= 0.23) model in agricultural lands of Qazvin Province, Iran. Moreover, SQI_a_ is simple and easier for mathematical calculations, while its limitation is that it relies on the subjective judgment of the researchers (Nabiollahi et al., 2017). Nabiollahi et al. (2018) evaluated SQ indices and showed that the SQI_w_ (*R* ^2 ^= 0.70) model was better than the SQI_a_ (*R* ^2 ^= 0.64) and SQI_n_ (*R* ^2 ^= 0.57), which is consistent with the results of our study.

### 4.4 Evaluation of soil quality under different grassland restoration measures

Grassland restoration measures had significant impacts on the SQI in the degraded desert steppe (**Figure 6**). Our results showed that SQI_w_-MDS values of grazing exclusion grassland (0.49) and reseeded grassland (0.57) were significantly higher than those of continuous grazing (0.34), indicating that short-term colonization of vegetation resulted in a rapid recovery of SQ in the study area. The grazing exclusion restoration policy of the SQ of degraded grassland is a long-term evolutionary process and requires a significant investment of both time and cost (Lu et al., 2015). In our research, it took 18 years for the grazing exclusion grassland to restore SQ, similar to that of the 3-year reseeding grassland. Although the SQI value of reseeding grassland was higher than the SQI value of grazing exclusion grassland, the difference between the values was not significant (**Figures 6A, C**). However, it was only 3 years since the continuous overgrazing of grassland changed to reseeding grassland, and the enhancement of SQ by planting native grass species was limited. In addition, the restoration rate of the SQI values of the 3-year reseeding grassland is five to seven times that of the 18-year grazing exclusion grassland.

Hence, our results verify that the ability of native grass species planting to enhance SQ may be close to or higher than the long-term grazing exclusion restoration management after 3 years of planting. As a consequence, considering the grassland soil restoration effect and short growth time of reseeding grassland in this study, native grass species reseeding may be another beneficial desert steppe restoration management in the study area and other arid regions. Overall, this study demonstrated that both grazing exclusion and reseeded restoration could improve SQ and that artificial reseeded restoration has a better capacity to recover SQ than natural grazing exclusion restoration in areas of Northwest China.

## 5 Conclusion

The grazing exclusion and reseeding restoration measures changed the soil physicochemical properties of degraded grasslands, which in turn significantly improved the soil quality of the semi-arid grazed degraded desert steppe in China. In addition, the fast restoration rate of SQI in reseeded grasslands indicates that the soil recovery rate of the reseeded grasslands was faster than the natural recovery of the grazing exclusion. The SQI_w_ used these MDS indicators to generate the most reliable SQI. Thus, the present study suggests that in the semi-arid desert steppe, natural recovery and reseeding are effective restoration measures for degraded desert steppe in the semi-arid area. We suggest that reseeding can recover soil quality faster in the short term. Moreover, the changes in soil quality after long-term reseeding need to be further elucidated by long-term monitoring, which will thereby enable us to identify the best measure to restore degraded desert steppe.

## Data availability statement

The raw data supporting the conclusions of this article will be made available by the authors, without undue reservation.

## Author contributions

HM conceptualized this study, supervised the writing, and revised the manuscript. QL led the writing. QL, YZ and JL performed the investigation and collected the data. HM and YS provided funding support. All authors contributed to the article and approved the submitted version.
